# A novel variant in *TLE6* is associated with embryonic developmental arrest (EDA) in familial female infertility

**DOI:** 10.1038/s41598-022-22687-y

**Published:** 2022-10-21

**Authors:** Mojdeh Akbari, Mehdi Mohebi, Katayon Berjis, Amin Ghahremani, Mohammad Hossein Modarressi, Soudeh Ghafouri-Fard

**Affiliations:** 1 Department of Medical Genetics, School of Medicine, Tehran University of Medical Sciences, Tehran, Iran; 2 Department of Medical Genetics, School of Medicine, Shahid Beheshti University of Medical Sciences, Tehran, Iran; 3 Department of Reproductive Biology, The Academic Center for Education, Culture and Research, Qom Branch, Qom, Iran; 4 Pars Human Gene Company, Tehran, Iran; 5 Department of Electrical Engineering, Sharif University of Technology, Tehran, Iran; 6 Urogenital Stem Cell Research Center, Shahid Beheshti University of Medical Sciences, Tehran, Iran

**Keywords:** Genetics, Molecular biology, Biomarkers

## Abstract

This study aims to identify genetic causes of familial female infertility characterized by embryonic developmental arrest (EDA) and repeated implantation failure (RIF) with oocyte donation IVF cycle. We used Whole-exome sequencing and Sanger validation to find causative genes in an Iranian consanguineous family that had 3 infertile daughters, 4 fertile daughters, and 2 fertile sons. All patients in this consanguineous family exhibited typical manifestations of unexplained RIF and EDA. Genetic analysis identified a homozygous missense variant (c.G1054C:p.G352R) in exon 13 of the *TLE6* gene that cosegregated with the EDA phenotype in an autosomal recessive pattern. Other members of the family, the gene carriers, remain clinically asymptomatic and fertile. Our findings identify a novel nonsynonymous variant, c.G1054C:p.G352R, in the *TLE6* gene within a consanguineous Iranian family with autosomal-recessive female infertility and broaden the genetic spectrum of *TLE6*-associated EDA.

## Introduction

Infertility is a common health problem defined as failure to conceive after one year of regular unprotected sexual intercourse. It is a notable reproductive health problem that affects more than 10% of people of reproductive age^1–3^. The technique of in vitro fertilization (IVF) is considered as a solution for a large number of infertile couples. Embryonic developmental arrest (EDA) is one of the several factors that can limit the success rate of this technique and is responsible for the high number of embryos that arrest during the first week of in vitro development. Approximately 10–15% of IVF embryos arrest in mitosis at the 2- to 4-cell cleavage stage and show no signs of cell death. Accumulating evidence indicates that this is a common phenomenon in humans. Approximately 10% of all human embryos created by IVF or intracytoplasmic sperm injection become permanently arrested in the early stages of cleavage in culture, and 40% of patients have at least one arrested embryo per treatment cycle^4^. The permanent arrest of embryos is a non-apoptotic event, since no morphological, no biochemical, and no molecular evidence of apoptosis has been observed before the 8-cell stage in bovine and human embryos^5–8^. Since the evaluation of the phenotype associated with EDA in any situation except recurrent IVF failure (RIF) is difficult and accessibility to it in the natural environment is not possible, the genetic cause of EDA in humans is not fully understood. According to previous studies, we can point to chromosomal abnormalities, and single-gene mutations in some genes such as *NLRP2*^9^*, NLRP5*^9^*, FBXO43*^10^*, PADI6*^11^*, Btg4*^12^*, CDC20*^13^*, MOS*^14,15^, and *ACTL7A*^16^ as the genetic causes of EDA. This process is also associated with aberrant regulation of several signaling pathways such as the Wnt and TGFβ signaling pathways^17^. In the stage of oocyte maturation, oocytes accumulate an essential amount of maternal RNAs and proteins that are used by the early embryo to zygotic genome activation (ZGA)^18,19^. Maternal factors regulate transcription, both directly and indirectly during ZGA^18^. Studies in mouse introduced *TLE6* as a fundamental maternal effect gene in embryonic preimplantation development^20,21^. It seems that *TLE6* has a pivotal role in symmetric cell division at the 2-cell stage. It stabilized the subcortical maternal complex (SCMC) which controls symmetric cell division in zygotes^22,23^. *TLE6*-null embryos have died at the cleavage stage^21,22^.

WD40-Repeat Proteins (WDR proteins) are one of the largest families encoded by humans^24^. They play important roles in many fundamental biological processes such as apoptosis^25^, DNA damage response^26^, protein degradation^27^, RNA processing^28^, transcription regulation^29,30^, histone modification^31^, and signal transduction^32^. WDR proteins often act as scaffolds to engage other molecules, forming protein–protein interactions or functional complexes^24^. They are classified broadly into 21 classes based on their domain architectures^24^. Transducin-like enhancer protein (TLE) family belongs to class 7 (TLE_N + WD40)^24,33^. One such WDR protein, TLE6, belongs to a TLE family that perform numerous critical functions such as controlling and regulating cell cycle progression, gene expression, post-translational modifications, and developmental process^33^. TLE6 has also been shown as a critical protein required in the embryonic process for female pregnancy. variants in the *TLE6* gene at the 19p13.3 locus (OMIM *612399) are responsible for autosomal recessive preimplantation embryonic lethality and EDA. To date, 13 variants have been identified^34–40^. The phenotypic spectrum seen in *TLE6* mutated patients in these reports suggested sex-associated and genotype–phenotype correlations. Females with bi-allelic variant that result in complete loss of TLE6 active site function exhibit infertility. In contrast, males and females with mono-allelic variant are fertile.

In this study, we characterized the phenotypic spectrum in a consanguineous Iranian family with autosomal recessive EDA due to variant in the *TLE6* gene. Here, we report three Iranian infertile sisters due to variant in *TLE6.* In-depth phenotyping of all members in this consanguineous family pinpoints a clear genotype–phenotype correlation between *TLE6* and EDA. We also report a novel variant [NM_001143986(*TLE6*): c. 1054 G>C (p. G352R)].

## Material and methods

### Subjects

One Iranian family segregating apparent RIF and EDA was ascertained for this study. Affected individuals underwent clinical examination. Whole blood samples were collected from all family members and genomic DNA was extracted, after obtaining written informed consent. This protocol has been approved by the ethics committee of the Shahid Beheshti and Tehran Universities of Medical Science, Tehran, Iran.

### Next-generation sequencing (NGS)

WES (Whole Exome Sequencing) for the family was performed, using the SureSelect XT V6 Human All Exon kit (Agilent Technologies, Santa Clara, CA, USA) following the manufacturer’s protocol. After library quantification and pooling, samples were sequenced on an Illumina NextSeq 500 System (Illumina, San Diego, CA, USA) using the Illumina V3 High Throughput kit. For 3 people in the family (II.11, II.16, II.18) library preparation, sequencing, and bioinformatics analysis were performed using illumina Platform**.**

### Segregation analysis

Segregation analysis was completed by Sanger sequencing on an ABI 3130 Sequencer (Foster City, CA, USA). All sequencing chromatograms were compared to published cDNA sequences for *TLE6* (NM_001143986.1), and nucleotide changes were detected using Finch TV Aligner.

### Ethics approval and consent to participant

All procedures performed in studies involving human participants were in accordance with the ethical standards of the institutional and/or national research committee and with the 1964 Helsinki declaration and its later amendments or comparable ethical standards. Informed consent forms were obtained from all study participants. The study protocol was approved by the ethical committee of Shahid Beheshti University of Medical Sciences and Tehran University of Medical Sciences. All methods were performed in accordance with the relevant guidelines and regulations.

## Result

### Bioinformatics analysis

Data quality was determined by FASTQC. Paired-end sequences were mapped to the human genome (UCSC hg19) using Burrows-Wheeler Aligner (BWA). Functional variant detection and annotation of genetic variants from high-throughput sequencing data were performed with the GATK (Genome Analysis Toolkit) and ANNOVAR software, respectively. Additionally, variants were filtered with MAF (minor allele frequencies) from the dbSNP, hapmap, Mutation Taster, Intervar, OMIM, Kaviar, 1000 Genomes, gnomAD, ExAC , and the Iranome. First, we filtered variants for quality (depth > 10 and quality score > 30) which was followed by minor allele frequency (MAF) (< 2%). Then, variants were prioritized based on variant-type (missense, nonsense, indel, or splice site), followed by *in-silico* prediction for conservation (GERP and PhyloP), and predicted deleteriousness (SIFT, PolyPhen2, and the CADD).

For extracting the candidate variants from genomic data of the affected (II.11, II.18) and their healthy sibling (II.16) we consider two hypotheses about infertility and embryonic developmental arrest (EDA) patients.

1. Variants that are associated with dominant diseases:
  All heterozygous variants are present in the patients and absent in the healthy controls.
2. Variants that are associated with recessive diseases:
  Homozygous variants are present in the patients and heterozygous or absent in the healthy controls.
  Compound heterozygous variants existing in the patients and missing in the healthy controls.
    $$Selected\;Variants=\bigcap_{i=1}^{2}Patients\left(i\right)-\bigcup_{i=1}^{1}Healthy (i)$$

By using this pattern, we found 7 variants in 7 genes (*WFS1, PLA2G7, IL17F, CHD4, A2ML1, CHRNA4, TLE6*). Six of them were placed in the first hypothesis, which we could not find any association for them with infertility and EDA, and one of them (*TLE6*) was placed in the second hypothesis, we found it in the homozygous format in the affected and heterozygous format in their healthy sister.

### Clinical characteristics of the patients

**Figure 1:**
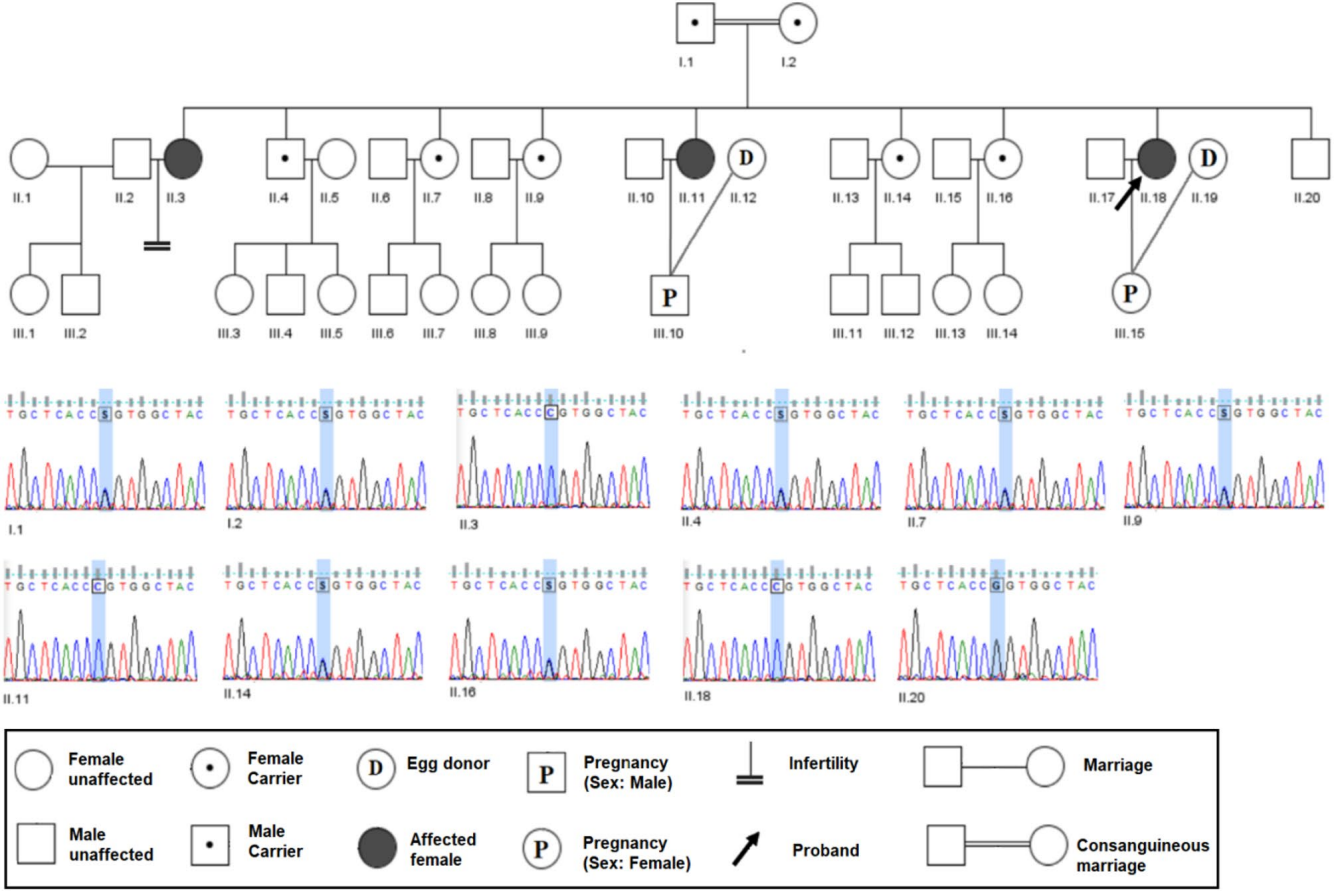
Pedigree of the studied family. Identification of *TLE6* variant in affected individuals and other family members. (*TLE6*: NM_001143986: exon13: c. G1054C: p. G352R).

The clinical information of the three patients carrying the biallelic *TLE6* variants are listed in Table 1, and their family pedigrees are shown in Fig. 1.

**Table 1:** Oocyte and embryo characteristics of the IVF and ICSI attempts of the female patients.

Patient	Age years	Age of marriage	Duration of infertility years	IUI	IVF/ICSI attempts with her oocyte	Outcomes	Total no. of PB1 oocytes retrieved	No. of fertilized oocytes	Viable embryos on day 3	IVF/ICSI attempts with oocyte donation	Outcomes after 2 embryo transfer in each time	Spouse information (Semen analysis)
II.3	56	17	31	3	3	All the embryos were arrested on day 3	1st.4	1st.2	0	0	0	Not available (But he got married and has 2 children)
							2st.5	2st.2	0			
							3st.9	3st.0	0			
II.11	37	21	15	1	3	All the embryos were arrested on day 3	1st.5	1st.3	0	3	1st. − 2st. − 3st.+	Sperm count: 88 Motility: 81.4% Morphology: 6% DFI TUNEL: 7% SCSA: 11%
							2st.5	2st.4	0			
							3st.4	3st.0	0			
II.18	32	21	10	1	3	All the embryos were arrested on day 3	1st.7	1st.2	0	3	1st. − 2st.− 3st.+	Sperm count: 97 Motility: 65% Morphology: 2% DFI: TUNEL: 4% SCSA: 10%
							2st.7	2st.0	0			
							3st.5	3st.0	0

All assessments including karyotypes, menstrual cycle assays, sex hormone levels, and transvaginal sonography revealed no abnormalities. Furthermore, their husbands also showed normal karyotypes and normal semen parameters (sperm morphology, motility, and sperm concentration) (Table 1). This Iranian consanguine family raised seven daughters. Three of them (II-3, II-11, and II-18) had infertility for several years. All the affected individuals underwent several IVF attempts. Two of the affected sisters (II-11 and II-18) even underwent three oocyte donation IVF attempts in the Reproductive Medicine Center.

The II-3 patients (56 years old) had undergone 3 unsuccessful IUI and 3 IVF/ICSI attempts. Totally 18 MII oocytes were retrieved in the three attempts. Only four oocytes were normally fertilized with two pronuclei (PN) zygotes, while the others were abnormally fertilized with 0PN or degradation on day 1. After cultivation, most of her embryos were arrested at the early stages with heavy fragmentation, and only two poor-quality embryos were available for transfer. Although the patient underwent 2 embryo transfer cycles, she failed to obtain a successful pregnancy. Her spouse got married and now has 2 children.

The other affected sister (II-11, 37 years old) underwent 1 unsuccessful IUI and 3 IVF/ICSI attempts with her oocytes and 3 IVF/ICSI attempts with donated oocytes. A total of 14 MII oocytes were retrieved in the 3 attempts. Some of her oocytes were abnormally fertilized with 0PN; 7 of them showed normal fertilization with 2PN zygotes on day 1. All of her embryos were arrested at the early stages having heavy fragmentation. Finally, after hysteroscopy and IVF with oocyte donation she got pregnant. In 3 IVF attempts with donated oocyte, 2 first attempts were failed and finally, in the last one, she got pregnant. Before the 3rd attempt, diagnostic hysteroscopy was performed on her and everything was normal. Her spouse's information is available on the Table 1.

The proband (II-18, 32 years old) underwent 1 unsuccessful IUI, 3 IVF/ICSI attempts with her oocytes, and 3 IVF/ICSI attempts with donated oocytes. A total of 19 MII oocytes were retrieved in the three attempts. Only 2 oocytes were normally fertilized with two pronuclei (PN) zygotes, while the others were abnormally fertilized with 0PN or degradation on day 1. After cultivation, all of her embryos were arrested at the early stages with heavy fragmentation. Totally, in 3 IVF attempts with donated oocyte, 2 first attempts were failed and finally, in the last one, she got pregnant. Before the 3rd attempt, diagnostic hysteroscopy was performed on her and everything was normal. Her spouse's information is available in the Table 1 and is completely normal.

### Identification of novel variant in *TLE6*

All patients in this consanguineous family exhibited typical manifestations of unexplained RIF and EDA. Genetic analysis identified a homozygous missense variant (c.G1054C:p.G352R) in exon 13 of the *TLE6* gene that cosegregated with the EDA phenotype in an autosomal recessive pattern (NCBI, ClinVar accession number is SCV002525878.). Other members of the family, gene carriers, remain clinically asymptomatic and fertile.

The homozygous variant [NM_001143986(*TLE6*): c.1054G>C (p. G352R)] was identified in the proband and all of her affected sisters. This variant was verified by Sanger sequencing. Both of the parents, four fertile elder sisters, and one elder brother were heterozygous carriers, indicating a recessive inheritance pattern (Fig. 1). This variant has not been reported in ClinVar, HGMD, gnomAD, Iranome, ExAC, dbSNP, and 1000G. Based on this situation this is a novel variant (Tables 2 and 3).

**Table 2:** Characteristics of the identified variant in the current study.

Variant allele	Homozygous
NM and location	TLE6:NM_024760:exon12:c.G685C:p.G229R, TLE6:NM_001143986:exon13:c.G1054C:p.G352R
start	2989593
cDNA alteration	c.G1054C
Protein alteration	p.G352R
Variant type	nonsynonymous SNV
ExAC (all/Asian)	Not found
gnomAD (all/Asian)	Not found
Iranome	Not found
SIFT	Damage
PolyPhen-2	Damage
Mutation taster	Damage
CADD-phred	25.2
varsome	VUS (PM2, PP3)
InterVar	VUS
Fraklin	VUS (PM2)
ClinVar	N/A
Hom f	N/A
AF	N/A
CADD	23.3
DANN	0.9977
Aggregated prediction	Uncertain

**Table 3:** Overview of *TLE6* variants described in EDA.

	Variant	Exon/intron	Inheritance	year	Ref	NCBI number
1	c.1529C>A (p.S510Y)	No data	Homozygous	2015	Alazami^40^	VCV000222026.2
2	c.1133delC, (p.A378Efs*75)	No data	Homozygous	2018	Xueqian Wang^39^	VCV000453260.1
3	c.1226G>A (p.Arg409Gln)	Exon 13	Homozygous	2020	Jing Lin^38^	Not available
4	c.1621G>A (p.Glu541Lys)	Exon 17	Homozygous	2020	Jing Lin^38^	Not available
5	c.388G>A (p.Asp130Asn)	Exon 7	Compound heterozygous	2020	Jing Lin^38^	Not available
6	c.1507G>A (p.Val503Ile)	Exon 15	Compound heterozygous	2020	Jing Lin^38^	Not available
7	c.541 + 1G>A	Intron7	Homozygous	2021	Bin Mao^34^	Not available
8	c.1245 − 2 A>G	Intron13	Homozygous	2021	Juan Liu^35^	Not available
9	c.1631_1632delCA (p.Pro544Argfs ∗ 5)	Exon 17	Homozygous	2021	Manyu Zhang^41^	Not available
10	c.475_476delCT, (p.Leu159Aspfs ∗ 14)	Exon 7	Homozygous	2021	Manyu Zhang^41^	Not available
11	c.222G>C, (p.Gln74His)	Exon 5	Compound heterozygous	2021/2021	Manyu Zhang^41^/Wei Zheng^37^	Not available
12	c.798_799insG, (p.Gln267Alafs ∗ 54)	Exon 12	Compound heterozygous	2021	Manyu Zhang^41^	Not available
13	c.893C>G, (p.Thr298Arg)	Exon 13	Homozygous	2021	Wei Zheng^37^	Not available
14	c.719C>G (p.Ala240Gly0	Exon 12	Homozygous and Compound heterozygous	2021	Wei Zheng^37^	Not available
15	c.1564G>C (p.Asp522His)	Exon 17	Homozygous	2021	Wei Zheng^37^	Not available
16	c.1013 G>A (p.Arg338His)	Exon14	Homozygous and Compound heterozygous	2021	Wei Zheng^37^	Not available
17	c. 1337G>A (p.Trp446*)	Exon 17	Homozygous and Compound heterozygous	2021	Wei Zheng^37^	Not available
18	c. 1054 G>C (p. G352R)		Homozygous	2022	Our study	Not available

### Results of conservative and in silico analysis

**Figure 2:**
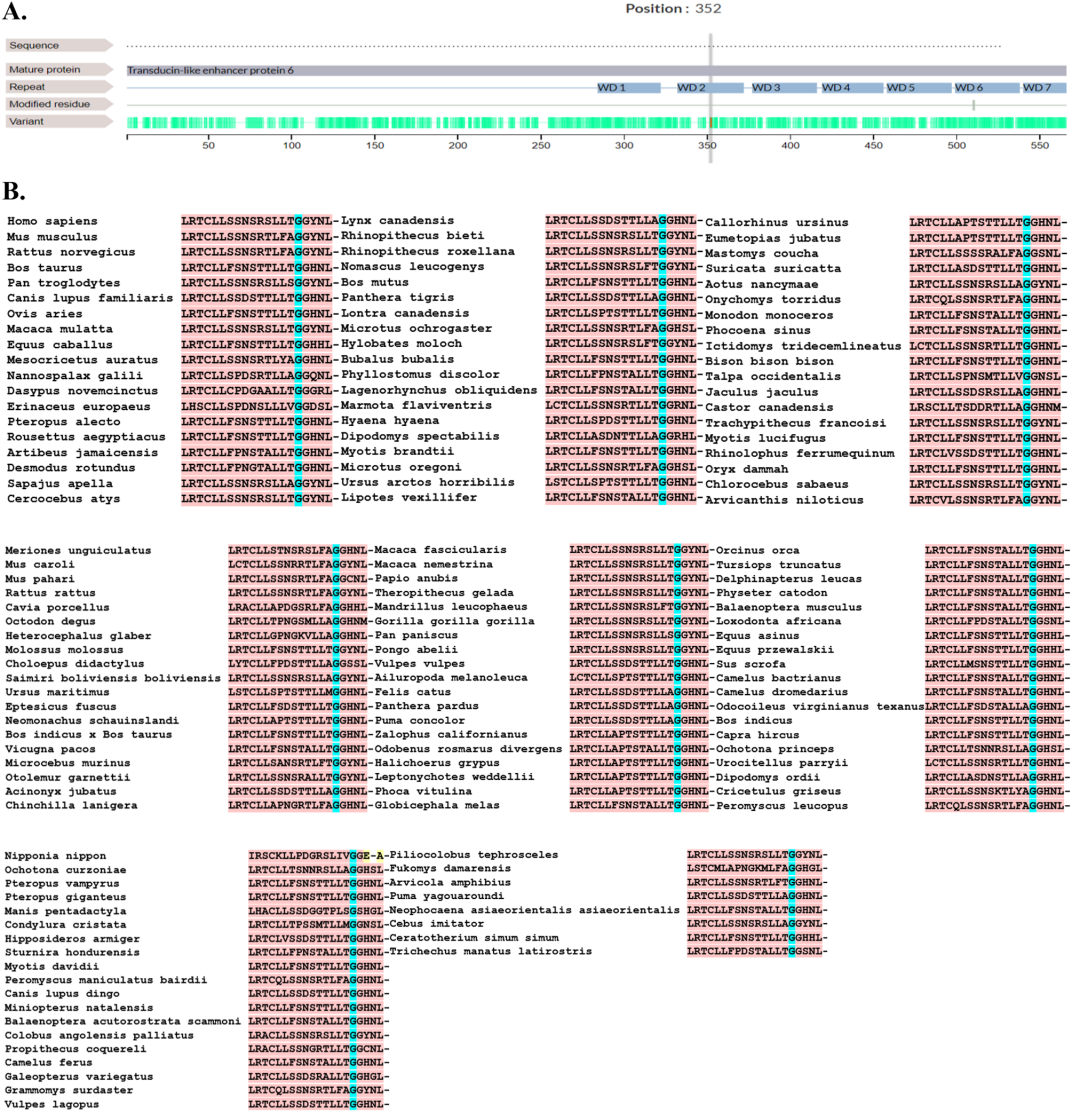
The locations and conservation of mutated residues in *TLE6* (*TLE6*:NM_001143986:exon13:c.G1054C:p.G352R). (**A**) The position of the variant is indicated in the genomic structure and protein structure of TLE6. (**B**) Conservation of mutated amino acids in TLE6 among 141 different taxonomies. The residue G352 is highly conserved across species.

**Figure 3:**
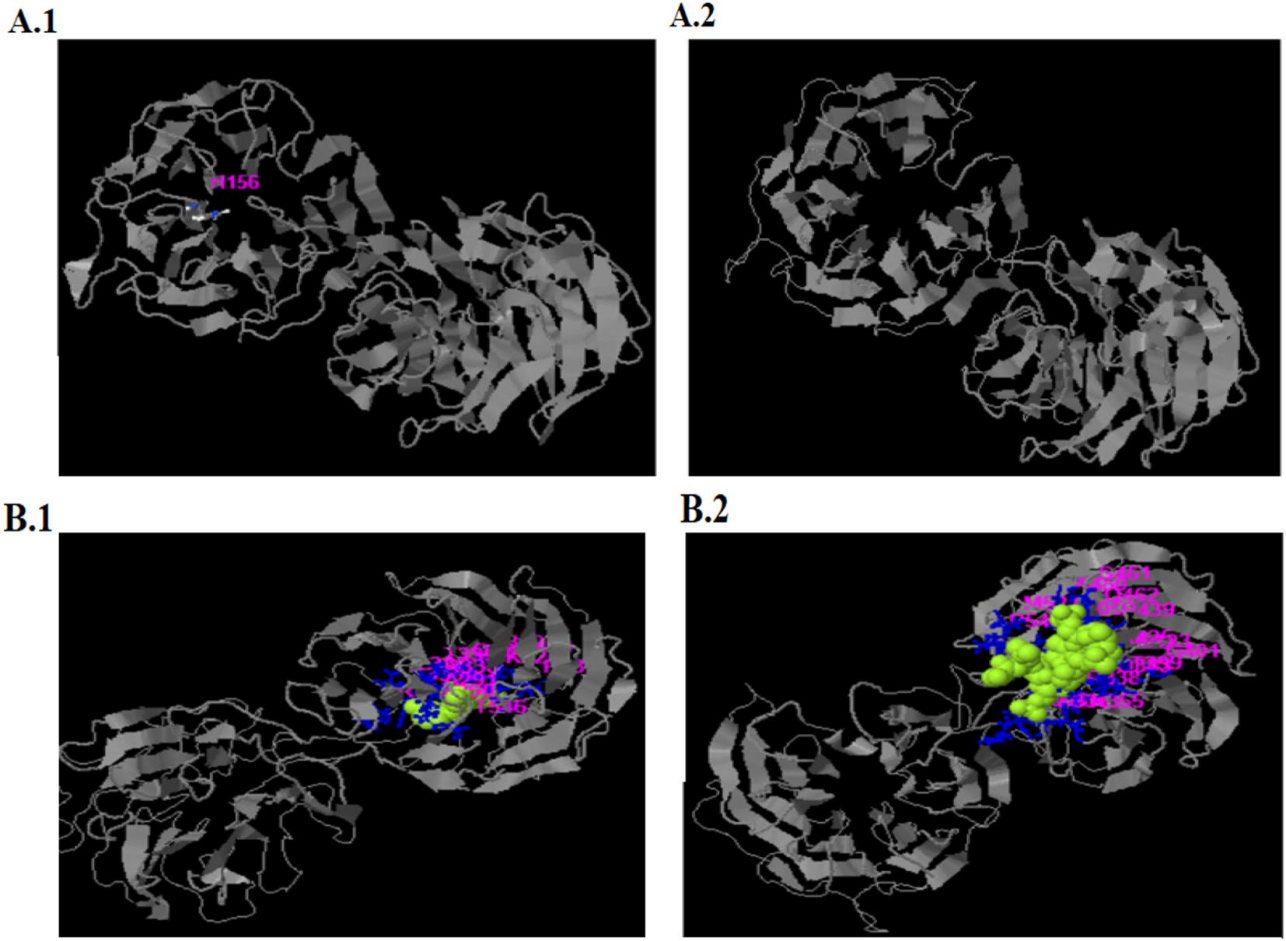
The overall effect of G352R variant on TLE6 structure, binding sites and active sites. (**A**) Comparison of mutant TLE6 binding sites with wild-type. (1. Wild-type/ 2. mutant). (**B**) Comparison of mutant TLE6 active sites with wild-type. (1. Wild-type/ 2. mutant).

The amino acids at position p.G352 of TLE6 were highly conserved in 141 different taxonomies, suggesting that the variant was likely pathogenic. The location of the *TLE6* variant and the conservation analysis among different species are shown in Fig. 2. The variant identified in this study (c.G1054C:p.G352R) resides in the buried residue of the WDR2 domain and seems to play a central role in the enzyme activity (Fig. 3). According to the three-dimensional (3D) structure of TLE6, the nonsynonymous variant caused a replacement of glycine with arginine at position 352, leading to the production of 30 new binding sites and deactivation of all of its active sites and 20 native binding sites compared with the Wild type (Fig. 3).

By using I-Taster, we found that TLE6 has 3 active site residues and 33 binding site residues. G352R variant gives rise to loss of all of its active site residues and 20 native binding site residues and achievement of 30 new binding sites, as a result, this variant caused TLE6 to have no active site and 43 binding sites (For more detail refer to attachments).

### Phenotypic spectrum of the patients with *TLE6* variant p.G352R

**Figure 4:**
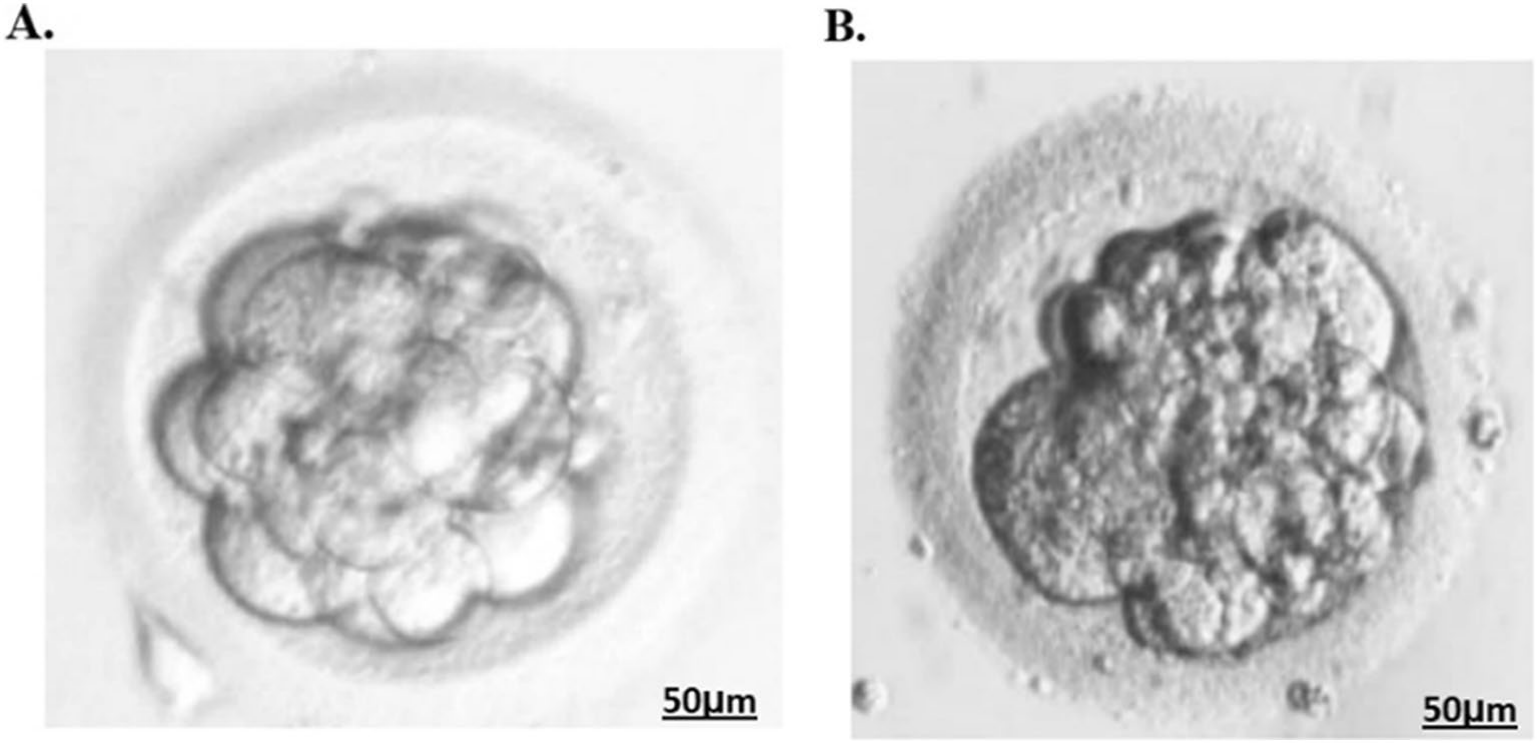
Phenotype of embryo from the proband (II-18).

We used light microscopy to observe the development and morphology of the embryos from family members II-5 and II-8 for 5 consecutive days in their last ICSI attempt. Five of the embryos on day 3 were arrested, whereas the others had a high percentage of fragmentation, and all of them failed to form blastocysts (Fig. 4).

The morphology of the embryo is derived from the control and the proband, respectively.

## Discussion

Many infertile couples have experienced recurrent IVF/ICSI failed attempts. In some of them, the obtained oocytes and ovulatory status look normal, but zygote formation and embryonic development are severely impaired. This study aims to identify genetic causes of familial female infertility characterized by EDA and RIF. We used whole-exome sequencing and Sanger validation to find causative genes in an Iranian consanguineous family that has 9 children, 3 infertile daughters, 4 fertile daughters, and 2 fertile sons. All patients in this consanguineous family exhibited typical manifestations of unexplained RIF and EDA. Genetic analysis identified a novel homozygous missense variant (c.G1054C:p.G352R) in exon 13 of the *TLE6* gene that segregated with the EDA phenotype in an autosomal recessive pattern. Other members of the family, gene carriers, remain clinically asymptomatic and fertile.

TLE (Transducin-like enhancer protein) family is a conserved family of corepressor proteins, which cannot bind DNA directly but repress transcription by interacting with partner proteins^33,42^. They repress gene expression through different mechanisms^43^. Two evolutionarily conserved domains are found in the TLE family: the carboxy-terminal WD40 repeat domain and an amino-terminal (TLE N-terminal), also known as the Q-rich domain. It seems that the Q-rich domain is important for oligomerization of TLE and binding to specific transcription factors and interactions with most transcription factors are mediated by the WD40 repeat domain^33,43,44^.

WD40-Repeat Proteins (WDR proteins) are one of the largest families encoded by humans^7^. They play important roles in many fundamental biological processes such as apoptosis^8^, DNA damage response^17^, protein degradation^18^, RNA processing^18^, transcription regulation^20,21^, histone modification^22^, and signal transduction^23^. WDR proteins often act as scaffolds to engage other molecules, forming protein–protein interactions or functional complexes^7^. They are classified broadly into 21 classes based on their domain architectures^7^. Transducin-like enhancer protein (TLE) family belongs to Class 7 (TLE_N + WD40)^7,24^.

There are four full-length *TLE* genes in humans named *TLE1-4* and three truncated forms, *TLE5-7*. The truncated *TLE* isoforms are thought to inhibit the function of the full-length TLEs (*TLE1-4*)^33^. *TLE6* is located on chromosome 19 at 19p13.3. It contains 17 exons that span more than 17 kb of genomic DNA and it encodes the Transducing-like enhancer protein 6 (Q9H808- TLE6- Human) with 572 amino acids. Expressing in different tissue such as the placenta, thyroid, lung, and testis. It has 7 WDR domains.

To date, 13 variants have been identified in *TLE6*—associated with EDA characterized by female fertility^34–40^. 64% of them were located in the WDR domain. This paper adds the 14th variant which is located in the WDR domain. The variant identified in this study (c.G1054C:p.G352R) leads to the replacement of Gly 352 with Arg residing in the buried residue of the WDR2 domain which plays a central role in enzyme activity. It is speculated that native *TLE6* has 3 Active site residues (156,125 and 145) and 33 ligand binding sites. This variant leads to missing all active sites and 20 native binding sites and also it leads to gaining 30 new binding sites.

By considering its roles in signaling pathways (Notch and Wnt)^45,46^ and some biological functions such as endoplasmic reticulum localization, spindle localization, mitochondrion localization, regulation of cell division, and regulation of transcription by RNA polymerase II, it would be clear that loss of its functions may lead to infertility.

In summary, this study extended the spectrum of genetic causes of familial female infertility characterized by EDA by reporting a novel variant in *TLE6*. Our result suggests oocyte donation as the best ART method for patients with biallelic *TLE6* variants right now.

## Data Availability

The datasets used and/or analyzed during the current study are available from the corresponding author on reasonable request. The identified variant in this study has been deposited into NCBI, ClinVar (accession number: SCV002525878) and will be publicly available per NCBI "Hold until publish" policy.
