# Author Correction: A novel variant in *TLE6* is associated with embryonic developmental arrest (EDA) in familial female infertility

**DOI:** 10.1038/s41598-022-26129-7

**Published:** 2022-12-13

**Authors:** Mojdeh Akbari, Mehdi Mohebi, Katayon Berjis, Amin Ghahremani, Mohammad Hossein Modarressi, Soudeh Ghafouri-Fard

**Affiliations:** 1 Department of Medical Genetics, School of Medicine, Tehran University of Medical Sciences, Tehran, Iran; 2 Department of Medical Genetics, School of Medicine, Shahid Beheshti University of Medical Sciences, Tehran, Iran; 3 Department of Reproductive Biology, The Academic Center for Education, Culture and Research, Qom Branch, Qom, Iran; 4 Pars Human Gene Company, Tehran, Iran; 5 Department of Electrical Engineering, Sharif University of Technology, Tehran, Iran; 6 Urogenital Stem Cell Research Center, Shahid Beheshti University of Medical Sciences, Tehran, Iran

Correction to: *Scientific Reports* 10.1038/s41598-022-22687-y, published online 21 October 2022

The original version of this Article contained an error in the spelling of the author Mohammad Hossein Modarressi, which was incorrectly given as Mohammad‑Hossein Modarresi.

The original Article has been corrected.

